# Enhancing Super-Resolution Network Efficacy in CT Imaging: Cost-Effective Simulation of Training Data

**DOI:** 10.1109/OJEMB.2025.3610160

**Published:** 2025-09-15

**Authors:** Zeyu Tang, Xiaodan Xing, Gang Wang, Guang Yang

**Affiliations:** ^1^ Department of BioengineeringImperial College London 4615 SW7 2AZ London U.K.; ^2^ School of Computing and Data EngineeringNingboTech University 199200 Ningbo 315104 China; ^3^ Department of Bioengineering and Imperial-XImperial College London 4615 SW7 2AZ London U.K.; ^4^ Royal Brompton Hospital 156726 SW3 6NP London U.K.; ^5^ School of Biomedical Engineering & Imaging SciencesKing's College London 4616 WC2R 2LS London U.K.

**Keywords:** Generative Al, synthetic models, super resolution

## Abstract

Deep learning-based Generative Models have the potential to convert low-resolution CT images into high-resolution counterparts without long acquisition times and increased radiation exposure in thin-slice CT imaging. However, procuring appropriate training data for these Super-Resolution (SR) models is challenging. Previous SR research has simulated thick-slice CT images from thin-slice CT images to create training pairs. However, these methods either rely on simplistic interpolation techniques that lack realism or on sinogram reconstruction, which requires the release of raw data and complex reconstruction algorithms. Thus, we introduce a simple yet realistic method to generate thick CT images from thin-slice CT images, facilitating the creation of training pairs for SR algorithms. The training pairs produced by our method closely resemble real data distributions (PSNR = 49.74 vs. 40.66, p $< $ 0.05). A multivariate Cox regression analysis involving thick slice CT images with lung fibrosis revealed that only the radiomics features extracted using our method demonstrated a significant correlation with mortality (HR = 1.19 and HR = 1.14, p $< $ 0.005). This paper represents the first to identify and address the challenge of generating appropriate paired training data for Deep Learning-based CT SR models, which enhances the efficacy and applicability of SR models in real-world scenarios.

## I. Introduction

Multi-slice helical CT enables precise, non-invasive disease analysis, but image quality—and thus diagnostic accuracy—depends heavily on acquisition thickness. Thin-slice scans (1.25 mm) offer richer radiomics detail and better differentiate benign from malignant pulmonary nodules than thick-slice scans (5 mm) [1], [2], [3]. However, storage and sharing constraints often limit PACS archives to lower-resolution images.

**Fig. 1.**
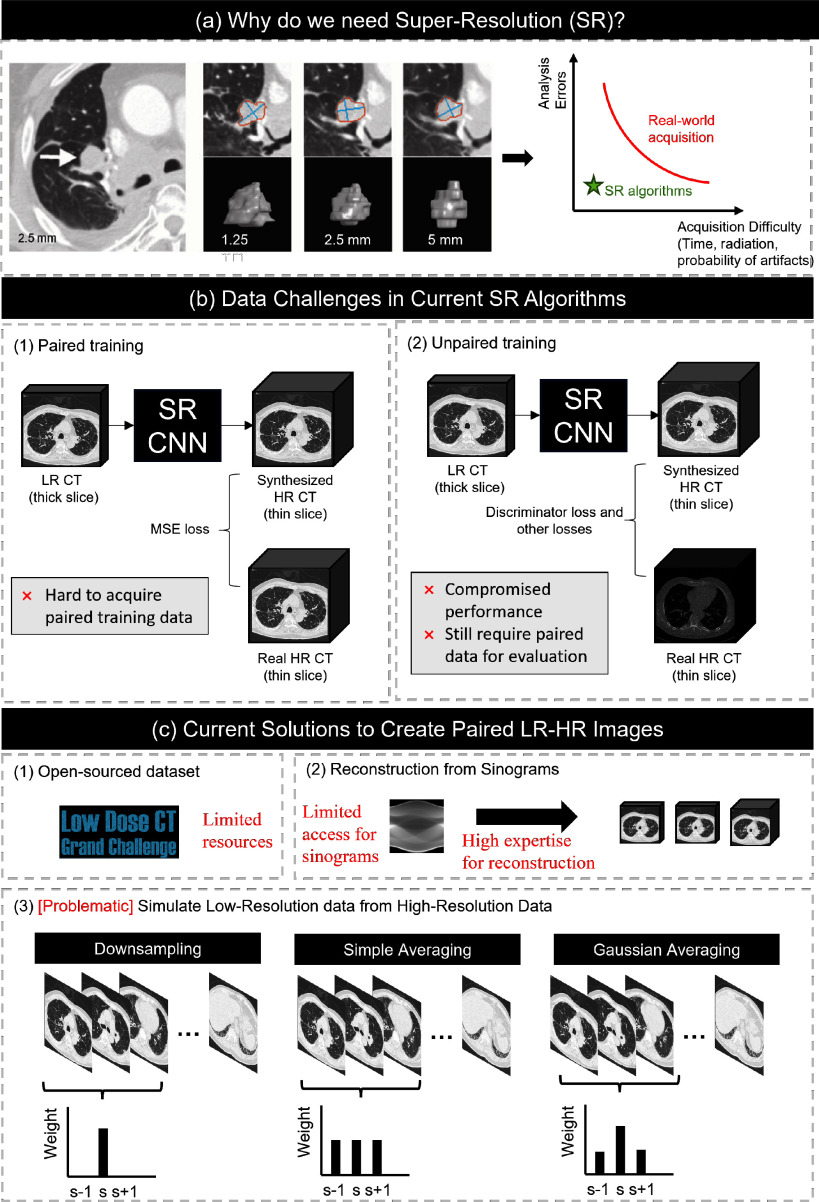
The image provides an overview of the necessity for our work. The rationale behind our work is to enhance the performance of CT super-resolution algorithms. (a) illustrates the need for SR in CT images; (b) highlights the difficulty in obtaining paired training data for SR CNNs, which can lead to compromised performance of the algorithms due to the lack of quality data for training and validation; (c) discusses the current methods used to simulate paired LR-HR images, pointing out the challenges and problems for failing to accurately represent the data.

This trade-off creates a gap between the high-resolution images needed for certain clinical tasks and what is typically available. Generative AI, particularly super-resolution (SR) methods using deep CNNs, can bridge this gap by converting low-resolution thick-slice CT images into high-resolution thin-slice equivalents, as shown in Fig. 1(a). However, training these models requires paired LR-HR data, which is rare, because routine scans generally do not acquire both resolutions simultaneously due to fixed scanning protocols.

The lack of publicly available paired LR-HR training data, along with limited acquisition practices, often compels researchers to simulate thick-slice CT images from existing thin-slice datasets [4], [5]. Those with access to high-resolution sinograms and in-house reconstruction tools may vary slice thickness by applying different reconstruction kernels [6]. However, most scanners use proprietary reconstruction methods, and access to raw sinogram data is rare. As a result, many researchers rely on basic techniques like spline interpolation [7] or slice removal [8] to approximate low-resolution images. These methods often ignore key factors like slice thickness and interval, leading to unrealistic image distributions and poor model performance on real-world, thick-slice CT scans.

This study sets out to introduce a novel simulation algorithm that can produce thick-slice CT images closely mirroring actual thick-slice scans, without the need for original sinograms or sophisticated reconstruction techniques. Our goal is to offer a nearly no-cost approach for generating paired LR-HR datasets that can support any CT super-resolution algorithm. As it stands, the AAPM-Mayo's LDCT dataset [9] is the only known public source of thin-thick slice pairs; hence, we used this dataset to validate our algorithm. We hypothesize that our simulation technique will generate thick-slice images that align more closely with real-life thick-slice images. Moreover, we propose that SR models trained with our simulated dataset will outperform those trained with other simulated datasets when applied to actual thick-slice images. The effectiveness of the SR models will be assessed using quantitative metrics including PSNR, MSE, SSIM and FID. Our research is the first to tackle the challenge of paired training data in the CT super-resolution domain.

## II. Related Works

### A. Conventional Simulation Methods

*Direct Downsampling (Nearest Neighbor):* Unlike interpolation-based methods, direct downsampling reduces the number of slices by removing slices according to a certain ratio to emulate a thicker slice. This technique can include taking every second, third, or nth slice to represent an increased slice thickness. For example, Ge et al. [10] simulated 1mm-3 mm and 1mm-3 mm CT image pair by performing direct downsampling on the thin-slice (1 mm) data. Wang et al. [11] generated thick slices by downsampling directly on the 1 mm thin-slice images. Wu et al. [12] simulated thick slices by downsampling directly on the 2.5 mm thin-slice images. To further adjust the results, Mansoor et al. [13] applied a 3D Gaussian smoothing filter on slices with 1 mm thickness followed by downsampling to create slices with 4 mm thickness on their in-house chest CT dataset.

*Simple Averaging (Linear Interpolation):* In simple averaging, pixel values from contiguous thin slices are directly averaged to generate a thick slice, assuming equal contribution from each slice. While easy to implement, this approach often leads to detail loss, as it ignores intensity distribution and structural relevance. For example, Park et al. [14] employed a method for thick-slice image simulation that involved averaging five slices with a 3 mm thickness to create a single slice with a 15 mm slice thickness on their in-house Brain CT dataset. In this approach, the middle slice was selected as the ground truth high-resolution image. Xie et al. [15] employed a method for simulating thick-slice images by averaging three or seven slices with a 1 mm thickness from their in-house brain CT dataset. This averaging process resulted in the creation of a single slice with a thickness of 3 mm or 7 mm, respectively.

*Gaussian Averaging (Gaussian Interpolation):* This technique involves the application of a Gaussian filter to the thin-slice images. The Gaussian filter gives more weight to the central slice and progressively less weight to the slices further away, based on the Gaussian distribution. This creates a smooth transition and can mimic the blurring effect seen in thicker slices. For example, Kudo et al. [7] simulated various combinations of slice thickness and slice interval by reducing the number of slices to either 1/4 or 1/8. They applied spline interpolation and random Gaussian noise to the reduced slices.

### B. Limitations

**Fig. 2.**
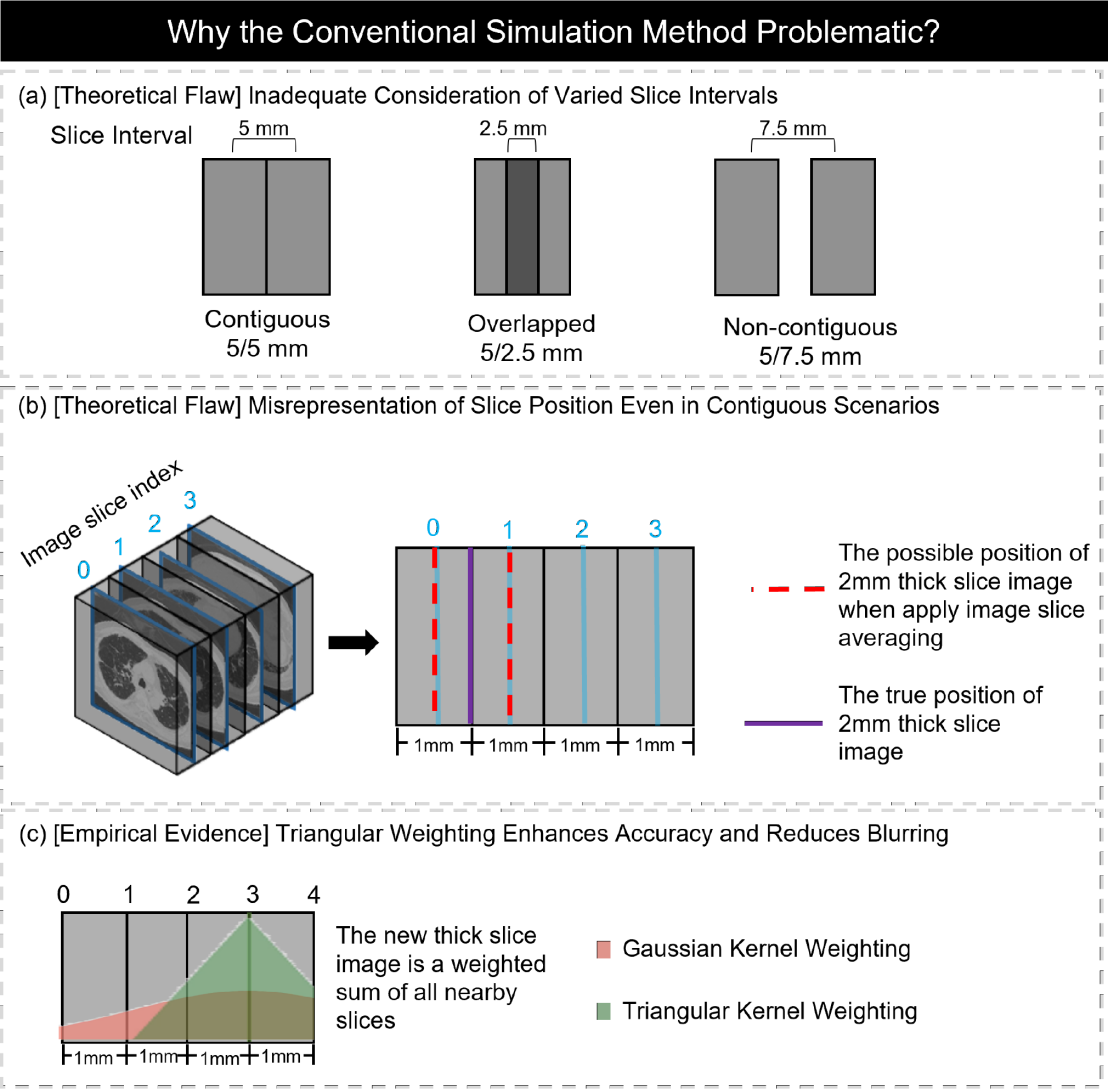
The limitations of conventional methods used for simulating thick-slice images from thin-slice CT scans. These traditional approaches can lead to inaccuracies in representing the true position (a), (b) and quality (c) of the thick-slice images.

Many conventional simulation methods assume that slice thickness is directly proportional to the number of slices—for example, reducing slice count to $n/3$ to simulate 3 mm slices from 1 mm slices. However, this assumption fails when slices are not continuous, as illustrated in Fig. 2(a). In the AAPM Mayo Clinic dataset, for example, patient L286 has 525 slices at 1 mm thickness but only 210 slices at 3 mm—not the expected 175—due to overlapping scan practices and 2 mm voxel spacing for the 3 mm images. This mismatch poses a critical issue: models trained to upscale z-slices by a fixed factor cannot accurately reconstruct real slice distributions, limiting their ability to generalize to actual clinical data.

Another theoretical shortcoming of conventional simulation algorithms is that they rely on image slice indexing, thereby disregarding the actual physical thickness of the images. For example, in Fig. 2(b), when simulating 2 mm thick images from 1 mm thick images, both Gaussian Averaging and Direct Downsampling algorithms select an existing slice as the “centre slice”. This results in the misplacement of the thick slice's position, as shown by the blue line, instead of its true position in real 2 mm thickness scans, indicated by the red line. This misplacement may lead to significant errors during the validation of SR performance on actual thick-slice datasets.

## III. Methods

### A. Position Correction

The proposed method for simulating thick-slice CT images from their thin-slice counterparts starts from determining the new positions for the simulated thick slices, utilizing the slice interval $d$. Different from the traditional interpolation techniques mentioned in Section II-B, we calculate the positions of the thick slices based on their actual physical locations according to The Patient-Based Coordinate System framework.

The spatial coordinates of each slice are documented in the DICOM header of the image. For a thin slice CT image, we first identify the coordinates of the starting $s$ and ending $e$ slices of the CT image volume. Then, the positions for the real thick slices are progressively calculated by adding increments of $d$, thus spanning the entire distance from $s$ to $e$. This approach ensures that the simulated thick-slice images are correctly placed in the physical space. The detailed procedure is outlined in Algorithm 1.

Algorithm 1: **Determine Slice Locations.**
**Input:** Start position $s$, End position $e$, Interval distance $d$
**Output:** Array of slice locations $L_{\text{thick}}$
1: Initialize $L_{\text{thick}}$ as an empty list
2: Set initial point $p \gets s$
3: **while** $s \leq p \leq e$ **do**
4: Add $p$ to $L_{\text{thick}}$
5: Update point $p \gets p + d$
6: **end while**

### B. Weighted Average

Following the determination of the axial coordinates for all thick slices with $p$ representing the z-coordinate of each thick slice, the next step is to compute the contribution from each thin slice to the reconstruction of a thick slice.

Mathematically, for each thick slice, we would like to compute the contribution of each thin slice, located at $l \in L_{\text{thin}}$, to the reconstruction of this thick slice whose position is noted as $p \in L_{\text{thick}}$.

Drawing inspiration from the Weighted Filter Back Projection (wFBP) [16], we have crafted a weighted sum algorithm designed to simulate thick-slice images from thin-slice counterparts. This is achieved through a triangular weighting function $g(p,l,t)$, with $t$ indicating the slice thickness. It should be noted that slice thickness $t$ is different from the slice interval $d$, as illustrated in Fig. 2, and we use the slice thickness to compute the contribution to better simulate the contribution from each sinogram slice to the image slice.

The function is mathematically defined as:

\begin{equation*}
g(p,l,t) = \max \left(0, 1 - \frac{{\left| p - l \right|}}{t}\right). \tag{1}
\end{equation*}

The generated thick slices are weighted sums of the thin slices, normalized by the total weight of the slices used. The detailed steps for the generation of thick-slice images are illustrated in Algorithm 2.

Algorithm 2: **Weighted Sum of Images for Thick Slices.**
**Input:** Thick-slice locations $L_{\text{thick}}$, Thin-slice images $\bm {I}_{\text{thin}} \in \mathbb {R}^{512\times 512\times d_{thin}}$, Slice thickness $t$
**Output:** Weighted sum images $\bm {I}_{\text{thick}}$
1: Initialize $\bm {I}_{\text{thick}}$ with a zero matrix $\mathit {O}\in \mathbb {R}^{512\times 512\times d_{thick}}$
2: **for** each location $l$ in $L_{\text{thick}}$ **do**
3: Initialize total weight $w_{\text{total}}$ to 0
4: Initialize weighted sum image $\tilde{i}$ to $\mathit {O}\in \mathbb {R}^{512\times 512}$
5: **for** each image $\bm {i}$ at location $p$ in $\bm {I}_{\text{thin}}$ **do**
6:
$w_{\bm {i}} \gets g(p,l,t)$

7: **if** $w_{\bm {i}} = 0$ **then**
8: Skip this iteration
9: **end if**
10: Accumulate weighted image: $\tilde{i} \gets \tilde{i} + \bm {i} \cdot w_{\bm {i}}$
11: Update total weight: $w_{\text{total}} \gets w_{\text{total}} + w_{\bm {i}}$
12: **end for**
13: Normalize $\tilde{i}$ by total weight: $\tilde{i} \gets \frac{\tilde{i}}{w_{\text{total}}}$
14: Append normalized $\tilde{i}$ to $\bm {I}_{\text{thick}}$
15: **end for**
16: **return** $\bm {I}_{\text{thick}}$

### C. Super-Resolution (SR) Models

To assess the quality of our simulated dataset for model training purposes, we chose four super-resolution models as benchmarks against our simulated thick-slice data, including VDSR [17], U-Net [18], ESRresnet [19], and ESRGAN [19]. It is important to clarify that while the primary target of the study is not to introduce new SR architectures, detailed implementation of these SR models are shown in the supplementary file.

## IV. Experimental Settings

### A. Dataset

This study primarily used two datasets: the TCIA LDCT-and-Projection dataset [20], containing 99 neuro (N), 100 chest (C), and 100 liver (L) scans, and the 2016 Low Dose CT Grand Challenge (LDCT-GC) dataset [9], which includes 30 contrast-enhanced abdominal CT scans with paired 1 mm and 3 mm reconstructions. Thick-slice data were simulated from the TCIA dataset for training, while the LDCT-GC dataset was used for testing.

### B. Evaluation Metrics

The Peak Signal-to-Noise Ratio (PSNR) and Root Mean Square Error (RMSE) are common metrics for evaluating image quality by comparing original and reconstructed images. PSNR measures the ratio between the maximum possible pixel value and reconstruction error, calculated as:

\begin{equation*}
\text{PSNR} = 20 \cdot \log _{10}\left(\frac{\text{MAX}_{I}}{\sqrt{\text{MSE}}}\right), \tag{2}
\end{equation*}

where $\text{MAX}_{I}$ is the maximum pixel value and MSE is the mean squared error:

\begin{equation*}
\text{MSE} = \frac{1}{mn} \sum _{i=0}^{m} \sum _{j=0}^{n} (I(i,j) - \hat{I}(i,j))^{2}. \tag{3}
\end{equation*}

RMSE, the square root of MSE, emphasises larger errors:

\begin{equation*}
\text{RMSE} = \sqrt{\text{MSE}}. \tag{4}
\end{equation*}

## V. Results

We assessed the performance of our proposed simulation method against Direct Downsampling, Simple Averaging, and Gaussian Averaging. The results from all conducted experiments, represented as mean $\pm$ standard deviation, are tabulated in this section. Unless otherwise specified, all results discussed here and in the following section are accomplished by simulating images with a thickness of 3 mm from those of 1 mm, utilizing the 2016 Low Dose CT Grand Challenge dataset.

This section proves our hypothesis that our method generates the most realistic training pairs for synthetic images. This results in the most accurate SR performance of all SR CNNs when testing on real thick-slice images.

### A. Image Fidelity Comparison

The results presented in Table I offer a detailed comparative analysis of various thick-slice simulation methods applied to two datasets from the 2016 Low Dose CT Grand Challenge, utilizing both the PSNR and the RMSE as the primary performance metrics.

**TABLE I:** Comparison Experiments on Different Thick-Slice Simulation Methods

**Dataset**	**Simulation Methods**	**PSNR**	**RMSE**	**SSIM**	**FID**
2016 LDCT-GC (D45)	Simple Averaging	40.6623 $\pm$ 3.1264$^{\dagger }$	0.0198 $\pm$ 0.0073$^{\dagger }$	0.9650 $\pm$ 0.0180$^{\dagger }$	0.0097 $\pm$ 0.0021$^{\dagger }$
	Gaussian Averaging	30.0160 $\pm$ 5.8123$^{\dagger }$	0.0800 $\pm$ 0.0590$^{\dagger }$	0.8870 $\pm$ 0.0312$^{\dagger }$	0.1239 $\pm$ 0.0162$^{\dagger }$
	Direct Downsampling	36.0757 $\pm$ 2.9024$^{\dagger }$	0.0334 $\pm$ 0.0131$^{\dagger }$	0.9489 $\pm$ 0.0204$^{\dagger }$	0.0243 $\pm$ 0.0050$^{\dagger }$
	Proposed	**49.7369 $\pm$ 2.5223**	**0.0068 $\pm$ 0.0020**	**0.9793 $\pm$ 0.0133**	**0.0074 $\pm$ 0.0034**
2016 LDCT-GC (B30)	Simple Averaging	42.0079 $\pm$ 2.8809$^{\dagger }$	0.0168 $\pm$ 0.0058$^{\dagger }$	0.9878 $\pm$ 0.0081$^{\dagger }$	0.0049 $\pm$ 0.0015$^{\dagger }$
	Gaussian Averaging	33.4766 $\pm$ 5.9684$^{\dagger }$	0.0553 $\pm$ 0.0464$^{\dagger }$	0.9360 $\pm$ 0.0243$^{\dagger }$	0.0660 $\pm$ 0.0090$^{\dagger }$
	Direct Downsampling	36.2342 $\pm$ 3.3025$^{\dagger }$	0.0334 $\pm$ 0.0150$^{\dagger }$	0.9711 $\pm$ 0.0119$^{\dagger }$	0.0164 $\pm$ 0.0018$^{\dagger }$
	Proposed	**48.5801 $\pm$ 7.3271**	**0.0108 $\pm$ 0.0099**	**0.9945 $\pm$ 0.0059**	**0.0020 $\pm$ 0.0018**

$^{\dagger }$ represents statistical significance (with Wilcoxon signed-rank test p-value $< 0.05$) compared with the proposed method.

The evidence strongly indicates that the proposed method outperforms Simple Averaging, Gaussian Averaging, and Direct Downsampling techniques in generating realistic thick slices from thin slices, applicable across both D45 and B30 reconstruction kernels. The validity of these superior results is supported by a Wilcoxon signed-rank test with a $p < 0.05$, signifying that the observed enhancements are statistically significant.

These findings corroborate our initial hypothesis, suggesting that the proposed simulation technique offers a more effective and accurate alternative for thick-slice simulations than conventional approaches.

### B. Image Utility as Training Dataset

We investigated our second hypothesis by training four different SR models using the data generated by top two simulation methods. According to Table II, we selected the Simple Averaging method for the utility comparison. After training the model on our simulated paired dataset, we tested these models' performance on true thick slice images to see if these models could reconstruct true thick slice images into thin slice images.

In each case, the performance of the SR models trained by the proposed simulation method outperforms those trained by the Simple Averaging simulation method, indicating improved image quality and lower error rates, respectively. The differences observed were statistically significant as determined by the Wilcoxon signed-rank test (p-value $< $ 0.05).

## VI. Discussions

### A. Visual Comparison of Fidelity

**Fig. 3.**
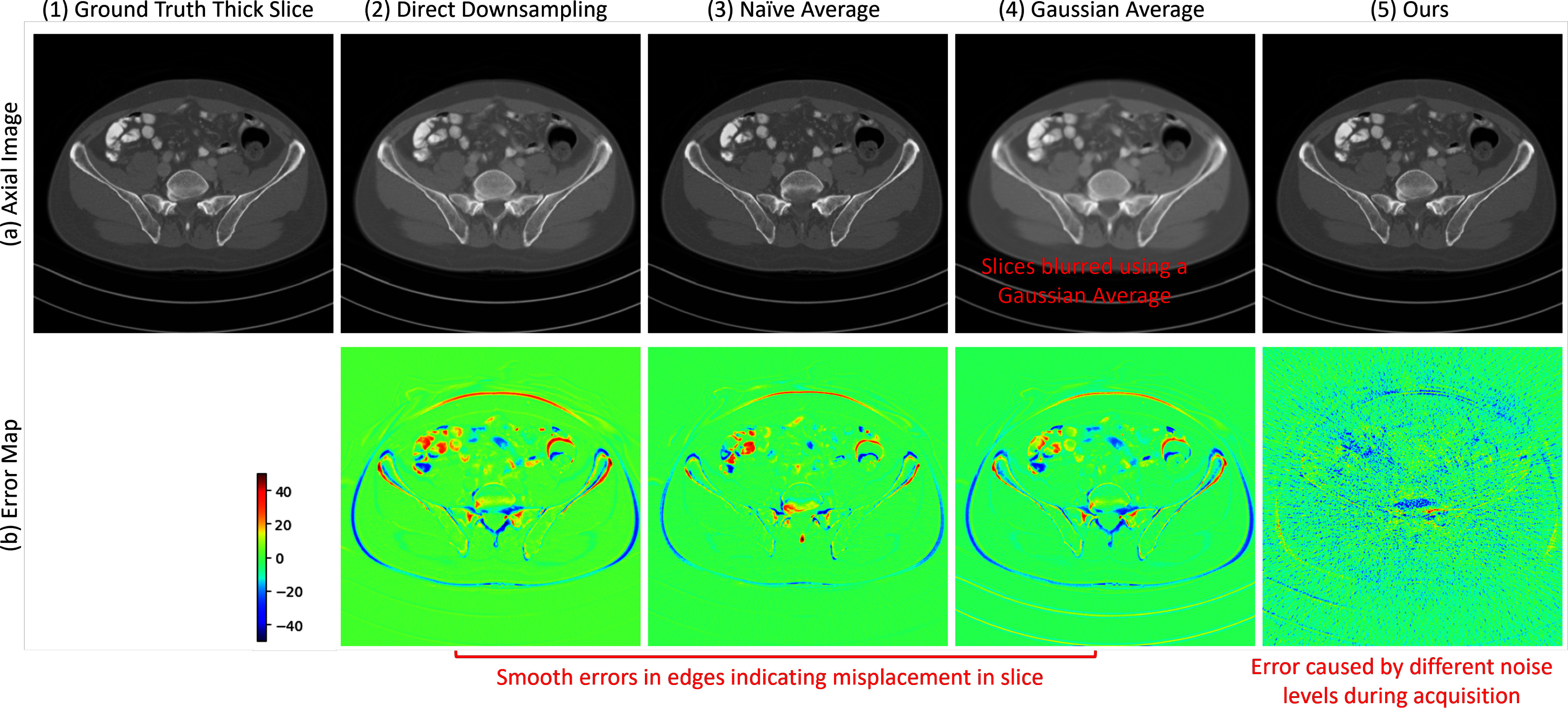
Example simulated thick slice using various simulation strategies and their corresponding error patterns. The Gaussian Average approach results in blurred imagery. The conventional simulations' error maps display smooth edge errors, indicating potential slice misalignment or displacement. In contrast, our method's error map demonstrates a markedly clearer outcome, with predominant noise patterns stemming from variations in slice thickness during acquisition and the Filtered Back Projection (FBP) process.

In this section, we provide visual assessments of simulated thick slice images across axial planes as shown in Fig. 3. We observed that traditional simulation methods consistently produce an error pattern, similar to motion-related artifacts. These error patterns are characterized by smooth, continuous errors along the image edges. Such patterns are not solely caused by slice misalignment but also by the averaging operations across slices, where the influence of adjacent slices is inaccurately estimated.

**Fig. 4.**
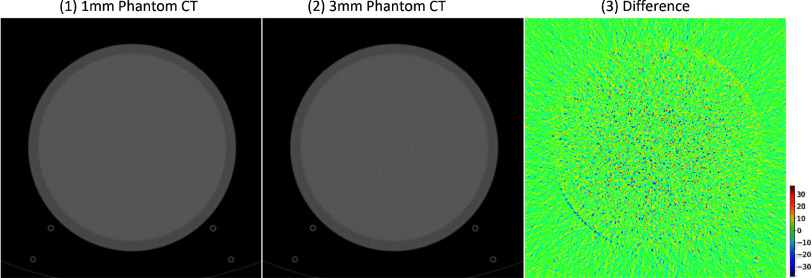
The full dose CT images reconstructed from B30 kernels acquired with different slice thickness and their differences.

Conversely, the error map for our proposed method demonstrates a more accurate simulation, with minimal processing errors. Moreover, we noted that noise levels present during image acquisition and the back projection process could introduce similar error patterns. Alshipli and Kabir's study [21] examined how slice thickness affects CT image noise, showing that thickness variations create distinct noise patterns. This is corroborated by the phantom images from the 2016 Low Dose CT Grand Challenge dataset, as depicted in Fig. 4. These images display a comparable error pattern as in Fig. 4(3), suggesting that our algorithm's failure to differentiate between thin and thick slice images primarily leads to noise-related errors. However, the impact of this noise is minimal, leading to only slight discrepancies.

### B. Visual Comparison of Utility

**Fig. 5.**
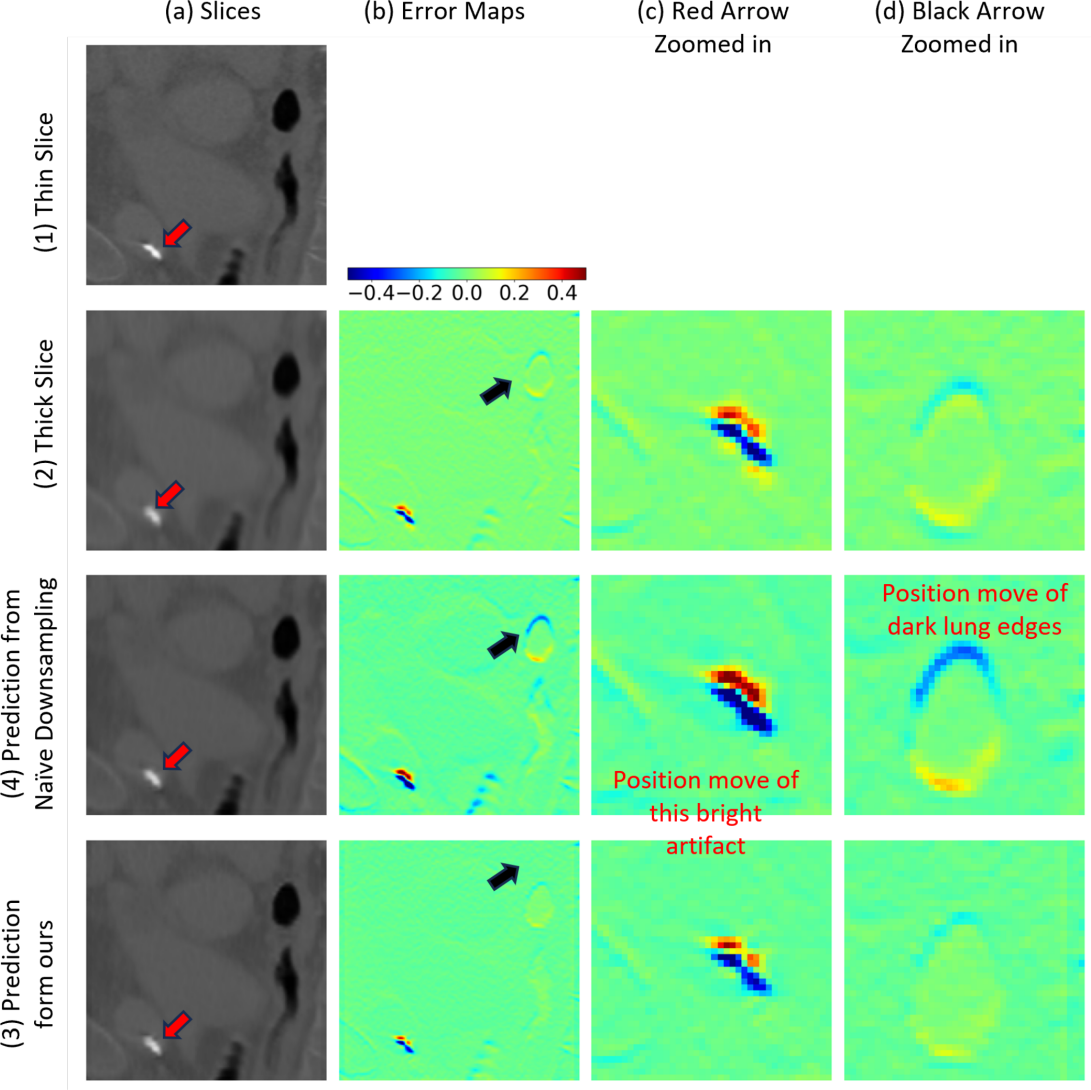
Super resolved slices using the ERSGAN model, which was trained on simulated data generated by a conventional naive method (3) and ours (4). The images are compared with the original thin (1) and thick (2) slices. The arrows highlight areas where our method's superiority is evident.

This section presents visual evaluations of super-resolved images (Fig. 5), generated by ERSGANs trained on data simulated using both our method and the conventional naive approach. ERSGAN was selected for its superior performance among the tested models. Thick-slice images were trilinearly interpolated to match thin-slice resolution for comparison.

As highlighted by the red arrows, thick slices exhibit blurred edge artifacts (2a). In super-resolved images trained on data from the naive downsampling method, these artifacts shift noticeably upward and to the right (4b), with edge displacement marked by black arrows. This misalignment indicates that poor simulation quality misguides SR model training, leading to inaccurate voxel mapping during reconstruction.

### C. Clinical Relevance

The primary clinical benefit of super-resolved CT images lies in their ability to facilitate accurate quantification analyses of anatomical structures, typically necessitating HRCT. To illustrate this, we applied an airway segmentation model [22], which was originally trained on HRCT, to both the original thick-slice CT images and super-resolved CT images produced by the ESRGAN model. Our goal was to evaluate the airway segmentation outcomes from these images to determine their efficacy in providing precise airway segmentation, thereby aiding the quantification analysis of thick-slice CT images.

The additional dataset used in the clinical relevance analysis is The Australian IPF repository (AIPFR), which has ethical approval from the Sydney Local Health District (protocol no. X14-0264). The diagnosis for each patient includes IPF, probable IPF, alternative diagnosis, and other fibrotic patterns based on the 2018 ATS/ERS/JRS/ALAT IPF guideline statement. We randomly selected 36 patients with an average slice thickness of 2.27 (STD = 0.44) mm. The average age is 72.09 (STD = 9.11).

The initial step involved applying ESRGAN models, trained on both naive and our simulation, to these thick-slice images, followed by airway segmentation inference. Subsequently, we filtered out radiomics features with low variance and selected five pertinent features relevant to mortality. The features we analysed include Elongation, the extent to which the shape of an ROI in the medical image is elongated; Maximum 2D Diameter Column, the largest possible diameter measured in the coronal plane of the airways; Maximum 2D Diameter Slice, the largest diameter measured in the axial plane of the airways; Mesh Volume, the volume of the airways based on a mesh approximation; and First order 10th Percentile, a statistical feature derived from the first-order intensity histogram of the airways. Multivariate analysis was then performed on these features, as presented in Table III.

**TABLE II:** Comparison Experiments on Models Trained by Different Simulated Thick-Slice Images

**Dataset**	**SR Model**	**Simulation Methods**	**PSNR**	**RMSE**	**SSIM**	**FID**
2016 LDCT-GC	VDSR	Simple Averaging	36.4722 $\pm$ 2.9112$^{\dagger }$	0.0320 $\pm$ 0.0131$^{\dagger }$	0.7617 $\pm$ 0.0649$^{\dagger }$	0.6344 $\pm$ 0.1071
(D45)		Proposed	**37.2176 $\pm$ 3.0876**	**0.0296 $\pm$ 0.0128**	**0.8140 $\pm$ 0.0640**	0.6407 $\pm$ 0.0977
	U-Net	Simple Averaging	36.5079 $\pm$ 2.9129$^{\dagger }$	0.0319 $\pm$ 0.0133$^{\dagger }$	0.7683 $\pm$ 0.0642$^{\dagger }$	0.6655 $\pm$ 0.1140
		Proposed	**37.9405 $\pm$ 2.1842**	**0.0262 $\pm$ 0.0071**	**0.8127 $\pm$ 0.0606**	0.7072 $\pm$ 0.1755
	ESRResNet	Simple Averaging	36.6322 $\pm$ 2.7383$^{\dagger }$	0.0312 $\pm$ 0.0120$^{\dagger }$	0.7859 $\pm$ 0.0650$^{\dagger }$	0.6407 $\pm$ 0.0659$^{\dagger }$
		Proposed	**37.8946 $\pm$ 2.5350**	**0.0267 $\pm$ 0.0094**	**0.8312 $\pm$ 0.0504**	**0.5556 $\pm$ 0.0701**
	ESRGAN	Simple Averaging	36.8371 $\pm$ 2.4522$^{\dagger }$	0.0301 $\pm$ 0.0098$^{\dagger }$	0.7831 $\pm$ 0.0598$^{\dagger }$	0.6535 $\pm$ 0.1118$^{\dagger }$
		Proposed	**37.9786 $\pm$ 2.4597**	**0.0264 $\pm$ 0.0089**	**0.8271 $\pm$ 0.0477**	**0.5563 $\pm$ 0.0876**
2016 LDCT-GC	VDSR	Simple Averaging	37.2458 $\pm$ 4.6983$^{\dagger }$	0.0323 $\pm$ 0.0209$^{\dagger }$	0.8630 $\pm$ 0.0404$^{\dagger }$	0.3894 $\pm$ 0.0281
(B30)		Proposed	**38.5657 $\pm$ 4.6613**	**0.0280 $\pm$ 0.0196**	**0.9060 $\pm$ 0.0315**	0.3919 $\pm$ 0.0201
	U-Net	Simple Averaging	37.4172 $\pm$ 4.4380$^{\dagger }$	0.0311 $\pm$ 0.0185$^{\dagger }$	0.8529 $\pm$ 0.0423$^{\dagger }$	0.3924 $\pm$ 0.0261
		Proposed	**39.7475 $\pm$ 3.5318**	**0.0226 $\pm$ 0.0112**	**0.9073 $\pm$ 0.0292**	0.4238 $\pm$ 0.0767
	ESRResNet	Simple Averaging	37.9217 $\pm$ 4.1180$^{\dagger }$	0.0289 $\pm$ 0.0171$^{\dagger }$	0.8788 $\pm$ 0.0379$^{\dagger }$	0.3258 $\pm$ 0.0374$^{\dagger }$
		Proposed	**40.2103 $\pm$ 3.9283**	**0.0223 $\pm$ 0.0150**	**0.9182 $\pm$ 0.0264**	**0.2608 $\pm$ 0.0220**
	ESRGAN	Simple Averaging	38.2401 $\pm$ 3.6225$^{\dagger }$	0.0270 $\pm$ 0.0136$^{\dagger }$	0.8629 $\pm$ 0.0391$^{\dagger }$	0.3086 $\pm$ 0.0446$^{\dagger }$
		Proposed	**40.5083 $\pm$ 3.8736**	**0.0215 $\pm$ 0.0144**	**0.9199 $\pm$ 0.0278**	**0.2331 $\pm$ 0.0187**

$^{\dagger }$ represents statistical significance (with Wilcoxon signed-rank test p-value $< 0.05$) compared with the proposed method.

**TABLE III:** Multivariate Cox Regression on Radiomics Features Extracted From Super-Resolved Images

Covariate	exp(coef)	exp(coef) Upper 95%	p-value
**Thick**			
Elongation	0	22.15	0.2
Maximum2DDiameterColumn	1.01	1.03	0.67
Maximum2DDiameterSlice	1.01	1.04	0.56
MeshVolume	1	1	0.82
First order 10Percentile	1.22	1.65	0.2
**Naive**			
Elongation	0	3.57E+06	0.38
Maximum2DDiameterColumn	1.04	1.15	0.36
Maximum2DDiameterSlice	0.94	1	0.04
MeshVolume	1	1	0.82
First order 10Percentile	0.76	0.93	0.01
**Ours**			
Elongation	4.05	2.52E+11	0.91
Maximum2DDiameterColumn	0.89	0.98	0.02
**Maximum2DDiameterSlice**	**1.19**	**1.31**	$\mathbf {< 0.005}$
MeshVolume	1	1	0.77
**First order 10Percentile**	**1.14**	**1.24**	$ \mathbf {< 0.005}$

Concordance = 0.85

The analysis revealed that only the features extracted from the super-resolved images from the model trained on our simulation algorithm correlated with mortality. This finding suggests that our algorithm holds significant potential for retrospective studies involving datasets acquired exclusively in thick slices.

### D. Limitations and Future Works

A key limitation of this study is its reliance on the AAPM-Mayo LDCT dataset—the only known public source of thin-thick slice pairs. As such, our method's performance has been evaluated mainly on this dataset, and its generalizability to patients with different health conditions remains uncertain.

We selected models based on their stability and suitability for 3D medical imaging, particularly their low GPU memory requirements. While other advanced 2D SR methods exist [23], [24], they were not included in this study. We implemented a slice-wise Denoising Diffusion Probabilistic Model (DDPM) [25] to enhance resolution on a 2D basis, but its performance fell short compared to full 3D methods. Future work will explore additional SR algorithms for 3D imaging.

To assess clinical utility, we applied segmentation models to super-resolved CT images to delineate anatomical structures like the airway tree [22]. However, limited annotations hindered the use of standard metrics like the DICE coefficient. We plan to expand evaluation to other clinical tasks and, with more detailed annotations, thoroughly analyse the utility [26] and accuracy of our method in clinical trial settings.

## VII. Conclusion

We present a simulation algorithm that enhances the performance of super-resolution (SR) models on real thick-slice CT images with minimal computational cost. Our method generates realistic thick-slice simulations, providing high-quality training data that improve SR outcomes on actual clinical scans.

The core innovation is a slice correction module that resolves misalignment issues common in traditional simulations, enabling accurate LR-HR training pairs. Additionally, inspired by Weighted Filtered Backprojection (wFBP), we introduce a targeted weighting system that applies a triangular weighting function to thin slices based on their position within the thick slice. This approach moves beyond simple averaging, prioritizing central slices to better mimic true reconstruction processes, reduce noise, and improve fidelity. By accounting for both slice thickness and increment, our method offers precise control over slice generation, surpassing the limitations of conventional techniques.

## Supplementary Materials

Supplementary Materials

## Conflict of Interest

All authors confirm that there are no conflicts of interest.

## Author Contributions

Z.T., X.X and G.Y. contributed to the conceptualisation of the study. Z.T. was responsible for the algorithm development and implementation. Analysis was conducted by Z.T. and X.X. The initial draft of the manuscript was written by Z.T. and X.X and G.Y., and Z.T., X.X., G.W. and G.Y. participated in the review and editing process.
