# Supplementary material for: Enhancing Super-Resolution Network Efficacy in CT Imaging: Cost-Effective Simulation of Training Data
Source: IEEE Open J Eng Med Biol. 2025 Sep 15;6:576–83. doi: 10.1109/OJEMB.2025.3610160 (PMC12599898; doi:10.1109/OJEMB.2025.3610160)
Supplement: Supplementary Materials [file supp1-3610160.pdf]

Supplementary Materials

Enhancing Super-Resolution Network Efficacy in CT Imaging: Cost-Effective Simulation of Training Data

Zeyu Tang, Xiaodan Xing, Gang Wang and Guang Yang, *Senior Member, IEEE*

I. Experimental Supplementary

A. Datasets

This study primarily used two datasets, and important features of these two datasets have been compiled and are presented in Table I and Table II for reference.

1) TCIA LDCT-and-Projection data: This compilation includes 99 neuro scans (denoted by N), 100 chest scans (denoted by C), and 100 liver scans (denoted by L). Half of each scan category comes from a SOMATOM Definition Flash CT scanner, a product of Siemens Healthcare from Forchheim, Germany. The remaining scans, consisting of 49 for the head, 50 for the chest, and 50 for the liver, were captured with a Lightspeed Volume Computed Tomography (VCT) CT scanner from GE Healthcare, based in Waukesha, WI. Some data in this collection might be utilized to reconstruct a human face. In order to protect the privacy of individuals involved, those accessing the data are required to sign and submit a TCIA Restricted License Agreement upon usage.

2) 2016 Low Dose CT Grand Challenge (LDCT-GC): The dataset consists of 30 deidentified contrast-enhanced abdominal CT patient scans, which were obtained using a Siemens SOMATOM Flash scanner in the portal venous phase. The data comprises two types: Full Dose (FD) data and Quarter Dose (QD) data. Full Dose data corresponds to scans acquired at 120 kV and 200 quality reference mAs (QRM), while Quarter Dose data refers to simulated scans acquired at 120 kV and 50 QRM. The provided dataset includes various components: 1) Projection data for all 30 patient scans, including 10 cases for training purposes (both FD and QD) and 20 cases for testing (QD only). 2) DICOM images for the 10 training cases, encompassing FD and QD data, with reconstructions using 1 mm thick B30 and D45 kernels, as well as 3 mm thick B30 and D45 kernels. 3) DICOM images for the 20 testing cases, consisting of QD data only, with the same reconstruction configurations as the training cases (1 mm thick B30 and D45 kernels, and 3 mm thick B30 and D45 kernels).

B. Super-Resolution (SR) Models

To assess the quality of our simulated dataset for model training purposes, we chose four super-resolution models as benchmarks against our simulated thick-slice data, including VDSR [1], U-Net [2], ESRresnet [3], and ESRGAN [3]. It is important to clarify that while the primary target of the study is not to introduce new SR architectures, detailed implementation of these SR models are shown in the 1.

Name	Structure	Our additional adaptation
VDSR	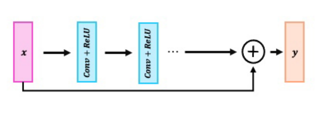	• Transformed 2-D operations to 3-D<br>• Reduced convolutional layers to 12<br>• Added global residual connection
U-Net	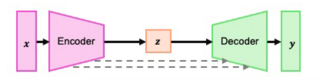	• Converted 2-D operations to 3-D<br>• added two residual blocks.
ESResNet	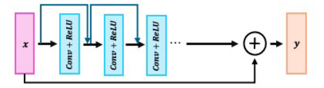	• Converted 2-D operations to 3-D.
ESRGAN	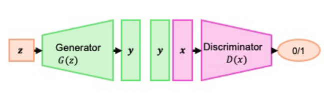	• Converted 2-D operations to 3-D<br>• Replaced original discriminator with patch-wise version.

Fig. 1. Illustration of our adaptations to four neural network architectures for super-resolution models

C. Implementation Details

The scripts used in this study were developed in Python3 and the PyTorch framework, and executed on Imperial College's HPC Clusters. Computations were run on an NVIDIA RTX 6000 GPU with 24 GB of memory. All SR models were trained over 1000 epochs using the Adam solver (learning rate = 1e-4) with batch size 32 and augmented with random horizontal flips during training. All models were trained using L2 loss to minimise errors. For ESRGAN, the adversarial loss was added to the L2 loss. The source code can be accessed at https://github.com/ayanglab/Thick2Thin.

II. Result Supplementary

A. Empirical Support on the Slice Correction

To clarify the rationale behind the slice correction step, we performed an analysis to quantify the similarity between thick slice images and their corresponding thin slice images by calculating the mean squared error (MSE) between them. The objective was to assess how a single thick slice image represents the cumulative information from all nearby thin slice images. This evaluation was designed to approximate how contributions from individual thin-slice images aggregate to form a specific thick-slice image.

Within this framework, Figure 2 presents the analysis focused on the first axial slice (starting from the top of the lung) in a low-resolution computed tomography (CT) series. We calculated the MSE between simulated 3mm thick slices and axial slices 2 to 6 from corresponding 1mm thick CT images. Figure 2(1) displays the axial slices for both the 1mm

TABLE I. Key data acquisition parameters for each exam type in TCIA LDCT-and-Projection-data

Scanner	Parameters	Head CT (N)	Chest CT (C)	Abdomen CT (L)
GE Healthcare (Discovery CT750i)	Tube Potential (kV)	120	80-120	80-120
	Contrast Enhanced	No	No	Yes
	Field of View	200-260	282-423	315-500
	Reconstruction Algorithm	Standard	Standard	Standard
	Thickness/Increment (mm)	5/5	1.25/1	5/3
Siemens Healthineers (SOMATOM Definition AS+, SOMATOM Definition Flash)	Tube Potential (kV)	120	120	100-120
	Contrast Enhanced	No	No	Yes
	Field of View	250	300-350	300-350
	Reconstruction Kernel	H40	B50	B30
	Thickness/Increment (mm)	5/5	1.5/1	5/3

TABLE II. Key data acquisition parameters for each exam type in 2016 Low Dose CT Grand Challenge

Reconstruction Kernel	Parameters	Full Dosage (FD)	Quarter Dosage (QD)
D45	Tube Potential (kV)	100-120	100-120
	Tube Current (mAs)	200	50
	Contrast Enhanced	Yes	Yes
	Thickness/Increment (mm)	1/0.8 and 3/2	1/0.8 and 3/2
B30	Tube Potential (kV)	100-120	100-120
	Tube Current (mAs)	200	50
	Contrast Enhanced	Yes	Yes
	Thickness/Increment (mm)	1/0.8 and 3/2	1/0.8 and 3/2

thin and 3mm thick slices, with slice spacings of 0.8mm and 1mm, respectively.

As is shown in Figure 2(1), the axial thin slice most similar to the first thick slice should be slice number 4 when the spacing is considered. Contrarily, simpler methods like direct downsampling and naive downsampling, which do not the physical locations, inaccurately designate slice number 3 as the most similar, based on a flawed assumption that mistakenly applies a factor of 3 to simulate the transition from 1mm to 3mm slices.

As in Figure 2 (2), our method, incorporating a physical space location correction based on the precise physical coordinates from the DICOM header, demonstrates the most authentic pattern of similarity to the thin slice images.

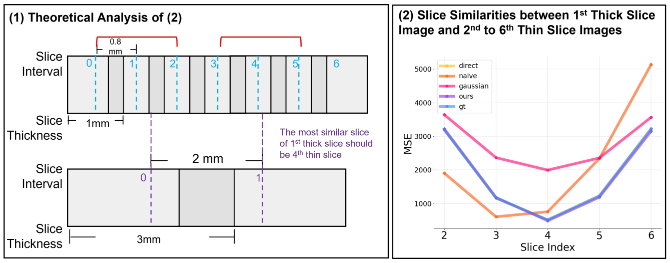

Fig. 2. Slice similarity comparison between thin slices and a selected simulated thick slice. As is shown, our proposed method simulates thick slices with the most realistic contributions from thin slices.

B. Benchmarking four different models

In our research, we conducted comparisons among various models to evaluate their SR effectiveness. The model that emerged as the most effective was the ESRGAN. This highlights the significant role of perceptual loss in SR models. For the benefit of other researchers, we have provided a summary of their efficiencies in Table III, serving as a preliminary guide. The models will be open-sourced in https://github.com/ayanglab/Thick2Thin.

TABLE III. The inference time, parameter size and performance of the 3D SR models implemented and refined by our paper. The performance is ranked by the mean value of the PSNR of these models on all testing datasets.

**Model Name**	**Inference Time (s/image)**	**Parameter Size (MB)**	**Avg Performance Rank**
VDSR	9	298	4
U-Net	6	4	3
ESRResNet	8	40	2
ESRGAN	8	83	1

References

[1] J. Kim, J. K. Lee, and K. M. Lee, "Accurate image super-resolution using very deep convolutional networks," in *2016 IEEE Conference on Computer Vision and Pattern Recognition (CVPR)*, 2016, pp. 1646–1654.

[2] O. Ronneberger, P. Fischer, and T. Brox, "U-net: Convolutional networks for biomedical image segmentation," in *Lecture Notes in Computer Science*. Springer International Publishing, 2015, pp. 234–241. [Online]. Available: https://doi.org/10.1007\%2F978-3-319-24574-4_28

[3] X. Wang, K. Yu, S. Wu, J. Gu, Y. Liu, C. Dong, Y. Qiao, and C. C. Loy, "ESRGAN: Enhanced super-resolution generative adversarial networks," in *Lecture Notes in Computer Science*. Springer International Publishing, 2019, pp. 63–79. [Online]. Available: https://doi.org/10.1007\%2F978-3-030-11021-5_5
